# Smoking Status in Pregnancy: A Retrospective Analysis in Northern Greece

**DOI:** 10.3390/jcm14020431

**Published:** 2025-01-11

**Authors:** Kyriaki Mitta, Ioannis Tsakiridis, Smaragda Drizou, Georgios Michos, Ioannis Kalogiannidis, Apostolos Mamopoulos, Chryssi Christodoulaki, Periklis Panagopoulos, Themistoklis Dagklis

**Affiliations:** 1 Third Department of Obstetrics and Gynaecology, School of Medicine, Faculty of Health Sciences, Aristotle University of Thessaloniki, 54642 Thessaloniki, Greeceamamop@auth.gr (A.M.); 2 Third Department of Obstetrics and Gynecology, University Hospital “ATTIKON”, Medical School of the National and Kapodistrian University of Athens, 12462 Athens, Greece; christodoulakichr@hotmail.com (C.C.); paninosrafaela@yahoo.gr (P.P.)

**Keywords:** smoking, pregnancy, cessation, risk factors, tobacco

## Abstract

**Background and Objectives:** Smoking has adverse effects on both maternal and fetal health and its incidence varies among different countries. The aim of this study was to identify the prevalence of smoking during pregnancy and to identify factors associated with smoking. **Materials and Methods:** This was a retrospective study conducted at the Third Department of Obstetrics and Gynecology, School of Medicine, Faculty of Health Sciences, Aristotle University of Thessaloniki, Greece, during an 11-year period (2013–2023). All women receiving antenatal care in our unit were eligible to participate when they attended the prenatal unit for the first trimester nuchal translucency scan (11^+0^–13^+6^ weeks). **Results:** Of the 12,074 pregnant women included in the study, 5005 (41.5%) reported themselves as smokers before pregnancy; the smoking cessation rate due to pregnancy was 70.2% (3516/5005) and the prevalence of smoking in pregnancy was 12.3% (1489/12,074). Multiparity was associated with less odds of smoking before pregnancy (OR: 0.79; 95% CI: 0.73–0.85), whereas advanced maternal age (OR: 1.17; 95% CI: 1.07–1.27) and obesity (OR: 1.44; 95% CI: 1.29–1.6) were associated with higher odds of smoking before pregnancy. Smoking prevalence in pregnancy was lower in women that conceived via assisted reproductive techniques (ARTs) (OR: 0.52; 95% CI: 0.38–0.70) and higher in cases of multiparity (OR: 1.12; 95% CI: 1.008–1.26) and maternal obesity (OR: 1.55; 95% CI: 1.20–2.00). Conception via ARTs was associated with higher odds of smoking cessation (OR: 1.9; 95% CI: 1.38–2.69), whereas multiparous (OR: 0.7; 95% CI: 0.62–0.8) and obese women (OR: 0.72; 95% CI: 0.61–0.85) were less likely to quit smoking. **Conclusions:** Pregnancy is a strong motivator for women to quit smoking, especially in primiparous women and those undergoing ARTs. Our findings highlight the need for more consistent smoking prevention and health promotion strategies in Greece as a very high proportion of women smoke before pregnancy and a substantial proportion continue in pregnancy.

## 1. Introduction

Smoking has been associated with detrimental effects on both maternal and fetal health, including miscarriage, congenital anomalies, stillbirth, small-for-gestational-age neonates, preterm birth, perinatal morbidity, and mortality [1,2,3]. Furthermore, with regards to long-term consequences, an increased risk of sudden infant death syndrome, as well as neurocognitive and developmental disorders, has been linked to smoking during pregnancy [4,5]. For these reasons, the World Health Organization advises against smoking during pregnancy, citing well-documented adverse obstetric and perinatal outcomes [6].

The prevalence of smoking during pregnancy varies across countries with similar economic levels. After 2015, smoking rates ranged from 4.2% in Sweden to 16.6% in France, with significant links to socio-economic factors [7]. In Canada, France, and the United States, smoking rates during pregnancy decreased among women with a higher socio-economic status, highlighting the disparities in maternal smoking within these countries [7]. Globally, the estimated prevalence of smoking during pregnancy was 1.7%; the European region reported the highest rates (8.1%), while the Eastern Mediterranean and African regions had the lowest (0.9% and 0.8% respectively) [8]. Most pregnant women who smoked during pregnancy were reported to be daily but light smokers [8].

Previously published data have shown that almost two-thirds of pregnant women cease smoking, probably motivated by their pregnancy status [9]. Native origin, nulliparity, and conception via assisted reproductive techniques (ARTs) have been identified as independent predictors of smoking cessation in pregnancy [9]. On the contrary, spontaneous conception and obesity have been identified as independent contributors to smoking during pregnancy [9].

Given the limited data on the prevalence of smoking during pregnancy in Greece, this study aimed to examine smoking rates among pregnant women and the potential factors associated with this addiction.

## 2. Material and Methods

This was a retrospective study conducted at the Third Department of Obstetrics and Gynecology, School of Medicine, Faculty of Health Sciences, Aristotle University of Thessaloniki, Greece, during an 11-year period (1/2013–12/2023). This unit is a tertiary referral center for a large area in northern Greece. All women receiving antenatal care in our unit were eligible to participate when they attended the prenatal unit for the routine first trimester nuchal translucency scan (11^+0^–13^+6^ weeks).

As part of standard prenatal care, a detailed obstetric and medical history was collected for each participant. This included maternal demographic factors (age, parity, and method of conception), lifestyle factors (smoking status and body mass index—BMI), and pregnancy-specific variables (type of gestation, history of pregnancy complications, etc.). Smoking status was categorized as ex- or current smokers, with further details on smoking cessation during pregnancy. Height and weight were measured at the first prenatal visit to calculate each patient’s BMI.

Women consented to the anonymity of their data and its potential use for future research purposes, with no incentives provided. Following the policy for observational studies that do not involve any interventions or modifications to routine patient care, no institutional board review was required for this study [10].

Participants were categorized based on maternal age (cut off: 35 years), parity (nulliparous or multiparous), method of conception (spontaneous or ARTs), and booking BMI (cut off: 30 kg/m²). Continuous variables were presented as mean ± standard deviations (SDs) and categorical variables as frequencies (percentages). The prevalence of smoking before and during pregnancy, along with the percentage of women who ceased smoking due to their pregnancy (quit smoking rate), were estimated. Univariate analyses of qualitative (dichotomous-categorical) variables were conducted using the chi-square test or Fisher’s exact test. A multivariate logistic regression model (enter method) was employed to identify factors independently associated with smoking status (current smoking, smoking before pregnancy, or smoking cessation, which were the dependent variables in each model). Estimated associations were reported as odds ratios (ORs) with 95% confidence intervals (CIs). Statistical significance was set at 0.05 and all analyses were carried out using the Statistical Package SPSS v. 28.0.

## 3. Results

Overall, 12,074 pregnant women attended the unit during the study period. The mean maternal age was 32 (SD: 5.2) and the mean booking BMI was 24.3 kg/m^2^ (SD: 5.0). Of these 12,074 pregnant women, 5005 (41.5%) reported themselves as smokers before pregnancy. Of these 5005 smokers, 3516 (70.2%) reported smoking cessation due to their pregnancy. Therefore, the overall prevalence of smoking in pregnancy was 12.3% (1489/12,074) (Table 1).

The univariate analysis revealed that the prevalence of smoking in pregnancy was lower in cases of ARTs and higher in multiparous and obese women, whereas maternal age and type of gestation were not associated with current smoking status. The multivariate logistic regression confirmed that the prevalence of smoking in pregnancy was lower in pregnancies conceived via ARTs (OR: 0.52; 95% CI: 0.38–0.70) and higher in multiparous (OR: 1.12l 95% CI: 1.008–1.26) and obese women (OR: 1.55; 95% CI: 1.20–2.00) (Table 2 and Table 3).

According to the univariate analysis, the prevalence of smoking prior to pregnancy was higher among nulliparous women, those aged more than 35 years old, and those with a BMI ≥ 30 kg/m^2^. The multivariate analysis confirmed that multiparity was associated with less odds of smoking before pregnancy (OR: 0.79; 95% CI: 0.73–0.85), while advanced maternal age (OR: 1.17; 95% CI: 1.07–1.27) and obesity (OR: 1.44; 95% CI: 1.29–1.6) were associated with higher odds of smoking before pregnancy (Table 2 and Table 3).

The prevalence of smoking cessation was higher in cases of ARTs, whereas it was lower among multiparous and obese women. The multivariate analysis confirmed that ARTs were associated with higher odds of smoking cessation (OR: 1.9; 95% CI: 1.38–2.69), whereas multiparous (OR: 0.7; 95% CI: 0.62–0.8) and obese women (OR:0.72; 95% CI: 0.61–0.85) were less likely to quit smoking (Table 2 and Table 3).

**Figure 1:**
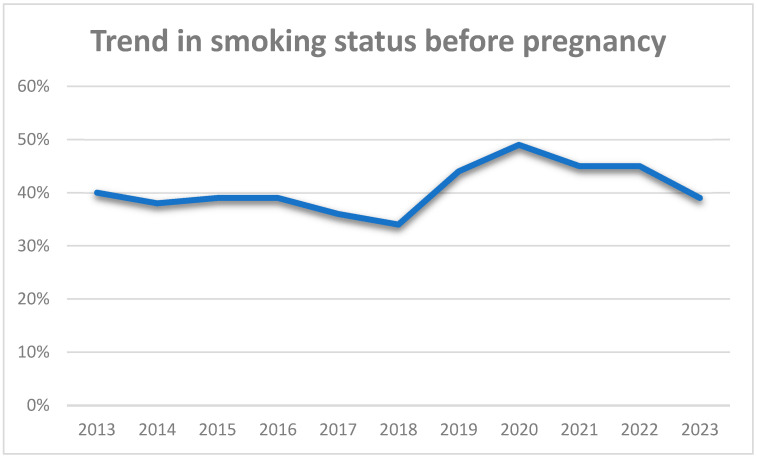
Timeline of smoking before pregnancy.

**Figure 2:**
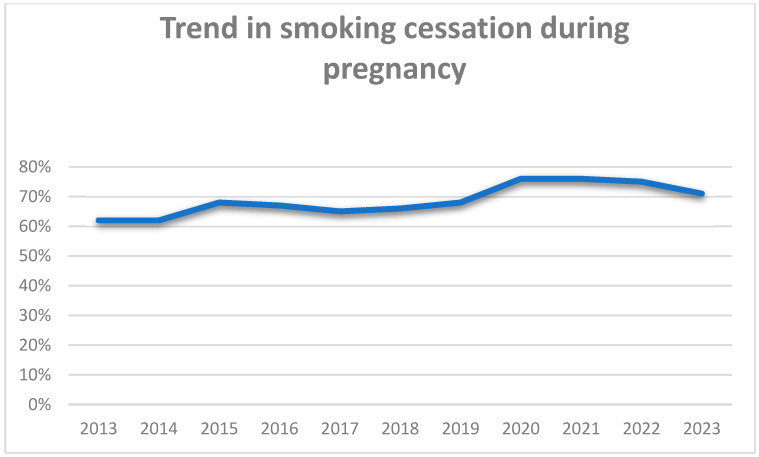
Timeline of smoking cessation rates due to pregnancy.

**Figure 3:**
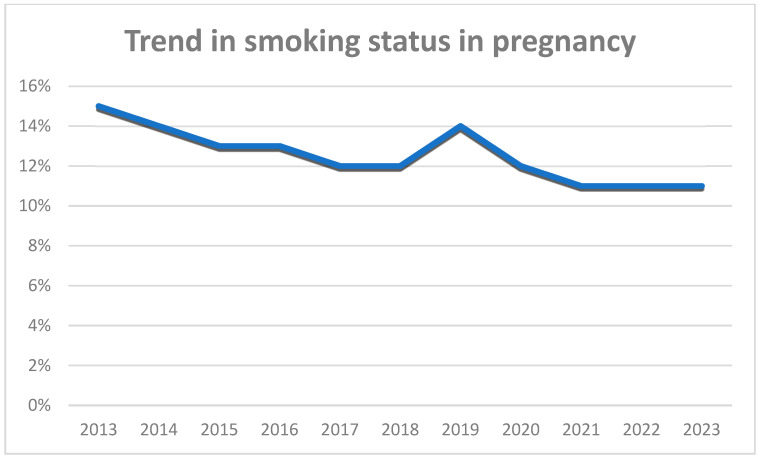
Timeline of smoking during pregnancy.

Regarding the time trend in smoking before pregnancy, the prevalence of smoking remained stable between 2013 and 2016, ranging from 38 to 40%, and then dropped in 2017–2018 and showed a sharp increase during the COVID-19 pandemic, with a tendency to return to pre-COVID levels, in 2023 (Figure 1). On the other hand, regarding the smoking cessation rates, the trend of smoking cessation remained stable until 2019, whereas during the pandemic, a marked increase in smoking cessation rates was observed (Figure 2). Regarding the time trend in smoking during pregnancy, the prevalence of smoking declined from 15% in 2013 to 11% in 2023 (Figure 3).

## 4. Discussion

The main findings of this study were as follows: (i) the prevalence of smoking before pregnancy was 41.5% and was higher among nulliparous, older, and obese women; (ii) the smoking cessation rate due to pregnancy was 70.2% and was higher in nulliparous women, and those with a lower BMI and those who conceived via ARTs; (iii) the rate of smoking during pregnancy was 12.3%, and multiparous, obese, and women with spontaneous conception were more likely to continue smoking in pregnancy; (iv) the prevalence of smoking before pregnancy was higher during the COVID-19 pandemic, while the smoking cessation rate also increased during the pandemic; and (v) the prevalence of smoking in pregnancy declined during the study period.

The prevalence of smoking among pregnant women is considered a public health issue due to its significant impact on the health of both the mother and the fetus [11]. More specifically, smoking during pregnancy has been linked to adverse outcomes such as reduced fetal growth, stillbirth, increased perinatal mortality, infant death, miscarriage, placental abruption, premature birth, accelerated lung aging, and chronic obstructive pulmonary disease [12,13,14]. Addressing this issue is critical, as jeopardizing the health of the mother and fetus can negatively influence the quality of life for families and society as a whole. Moreover, these outcomes have profound psychological, socioeconomic, and social ramifications, with the potential to adversely affect future generations.

This study showed that smoking in pregnancy was reported to be 12.3%, in a large sample of unselected pregnant women in northern Greece. This rate is lower than what has previously been reported in the Greek population; in a previous study, the rate of smoking during pregnancy was 13.2%, which was quite similar, yet slightly higher [9]. Of note, this is the largest study conducted in pregnant women in Greece and the smoking rate was comparable to those reported by other European countries [15]. Similarly, high rates of smoking during pregnancy have been previously reported in Romania, where low socioeconomic status was identified as an independent contributor of smoking in pregnancy [16]. The prevalence of smoking during pregnancy has raised significant concerns among researchers, prompting investigations into potential social and demographic predictors on an international scale. Women who smoke during pregnancy are more likely to have a low income, a single-parent status without a partner or social support, and reliance on maternity benefits provided by the state; they also have poor educational levels, experience negative judgment from their social environment, and face a heightened risk of depression and other psychiatric disorders during pregnancy [17,18,19].

According to the literature, the type of living regions seems to have an impact on smoking trends; living in rural areas has been associated with lower smoking prevalence that does not change during pregnancy [1,20]. A recent study that included Greek pregnant women, living in a large city, revealed a significantly increased rate of smoking during pregnancy compared to pregnant women living in rural areas (19.7% vs. 17%) [1,21]. The present study indicates a decrease in smoking prevalence in the wider area of northern Greece; however, geographical trends of smoking prevalence may exist. Data from the United Kingdom indicate a declining trend in the prevalence of smoking during pregnancy over time, with the most significant reduction occurring between 2005 and 2015, reaching 11%; from 2015 onward, the rate of reduction slowed down [22]. Similarly, in our dataset, the greatest decline occurred up to 2015, after which the prevalence stabilized.

Spontaneous rather than conception via ARTs was associated with higher odds of smoking during pregnancy, a finding that agreed with other studies [9]. Multiparous women were also more likely to smoke during pregnancy compared to nulliparous, which was in accordance with the results of a study of Puerto Rican pregnant women; multiparous women were more than twice as likely to smoke in pregnancy (OR: 2.1; 95% CI: 1.4–3.2) and in accordance with the results of a study on Greek pregnant women [21,23]. According to previously published data from our unit, the prevalence of smoking during pregnancy was higher among obese women (BMI ≥ 30 kg/m^2^), which was in accordance with the findings of the current study [9]. Several studies have suggested that smoking behavior is significantly related with body weight and obesity [24,25,26,27]. Regarding parity, the literature suggests that multiparous women are less likely to smoke before pregnancy (OR: 0.77; 95% CI: 0.67–0.89), which is in accordance with our findings [9].

Regarding smoking cessation rates, there is a wide range reported in the literature, which varies from 42.5% to 63.4% [9,28,29,30]. The rate of smoking cessation was higher in our study (70.2%) than that reported in the literature, indicating that pregnancy is a significant motivator among Greek women. Smoking cessation rates are reported to be higher among women conceiving via ARTs [31], which is consistent with our findings. Numerous reports indicate that morbidly obese individuals often exhibit a compulsion for unhealthy food and smoking [32], which reasonably explains our findings that women with higher BMIs have fewer odds of smoking cessation. Nulliparous women were more likely to quit smoking during pregnancy compared to multiparous, according to our findings, which was in agreement with the literature [33].

The increased prevalence of smoking during the pandemic could be reasonably explained by the psychological distress at that period. The data regarding smoking trends during the pandemic are conflicting [34,35], with several studies supporting its increase during that period [36,37]. Data arising from United Kingdom support the notion that smoking cessation rates in pregnant women and the demand for antenatal smoking cessation services were unchanged during the COVID-19 pandemic [38]. Moreover, data arising from the Greek pregnant population during the pandemic reveal that nicotine consumption was reduced in pregnancy, which was in agreement with the findings of our study [39]. A combination of healthcare support differences, cultural attitudes, pandemic-related lifestyle changes, and economic or psychological stressors probably contributed to the varied responses among pregnant women in the UK and Greece.

The present study has certain limitations; the information regarding smoking was based on self-reported data from constructed questions on smoking habits without using advanced validated psychometric instruments. Furthermore, the retrospective study design is considered as a limitation. Our findings are based on a sample of pregnant women in northern Greece, so the results cannot be generalized to the whole Greek population; however, this is the largest Greek study on smoking in pregnancy. Finally, this study did not assess for potentially important confounders i.e., income, educational and employment status, and passive smoking.

## 5. Conclusions

Pregnancy is considered an optimal time for smoking cessation interventions; pregnant women are highly motivated to stop smoking and have frequent and regular antenatal visits, which provides multiple opportunities to assess and promote cessation strategies. Furthermore, concerns over the dangers of cigarette smoking for the fetus serve as an additional motivator to stop smoking. However, smoking during pregnancy still remains widespread in numerous countries. Our findings highlight the necessity for additional studies concerning the impact of smoking on perinatal outcomes, the initiation of measures to create cessation programs before conception, and an assessment of effective interventions to promote a smoke-free environment during pregnancy. Our results should guide efforts in smoking prevention initiatives and health promotion strategies, while also highlighting the importance of improved accessibility to smoking cessation programs for expectant mothers.

## Figures and Tables

**Table 1:** Sociodemographic and obstetric characteristics of pregnant women attending the Fetal and Maternal Unit for a routine first trimester ultrasound (*n* = 12,074).

Characteristics	Frequency	Percentage (%)
**Maternal Age (Years)**		
<35	8667	71.8%
≥35	3407	28.2%
**Parity**		
Nulliparous	6442	53.4%
Multiparous	5632	46.6%
**Method of conception**		
Spontaneous	11,373	94.2%
ARTs	701	5.8%
**BMI (kg/m^2^)**		
<30	10,502	87%
≥30	1572	13%
**Smoking before pregnancy**		
No	7069	58.5%
Yes	5005	41.5%
**Current smoker**		
No	10,585	87.7%
Yes	1489	12.3%
**Smoking cessation due to pregnancy**		
No	8558	70.8%
Yes	3516	29.1%
**Type of gestation**		
Singleton	11,725	97.1%
Twins	349	2.9%

ARTs: assisted reproductive techniques; BMI: body mass index.

**Table 2:** Factors associated with smoking among pregnant women in Northern Greece—Univariate analyses.

Explanatory Variables	Current Smoking	Smoking Before Pregnancy	Smoking Cessation
	Univariate Analysis		
	OR (95% CI)		
Mode of conception (ARTs vs. spontaneous conception)	0.54 (0.4–0.72) ***	1.01 (0.87–1.18)	2.08 (1.53–2.83) ***
Twins (singleton vs. multiple gestation)	0.83 (0.59–1.18)	1.1 (0.91–1.4)	0.73 (0.5–1.06)
Parity	1.2 (1.09–1.35) ***	0.8 (0.77–0.89) ***	0.66 (0.59–0.75) ***
Maternal age	1.1 (0.97–1.24)	1.1 (1.03–1.21) *	1.02 (0.9–1.17)
BMI	1.66 (1.44–1.92) ***	1.4 (1.2–1.5) ***	0.69 (0.59–0.81) ***

ARTs: assisted reproductive techniques, BMI: body mass index, OR: odds ratio, CI: confidence interval, MD: mean difference. Current smoking: yes/no, smoking before pregnancy: yes/no, smoking cessation: yes/no. *: *p*-value < 0.05; ***: *p*-value < 0.001.

**Table 3:** Factors associated with smoking among pregnant women in Northern Greece—Multivariate analyses.

Explanatory Variables	Current Smoking	Smoking Before Pregnancy	Smoking Cessation
	Multivariate Analysis		
	OR (95% CI)		
Mode of conception (ARTs vs. spontaneous conception)	0.52 (0.38–0.70) ***	0.861 (0.72–1.02)	1.9 (1.38–2.69) ***
Twins (singleton vs. multiple gestation)	1.06 (0.73–1.53)	1.12 (0.89–1.4)	0.99 (0.66–1.48)
Parity	1.12 (1.008–1.26) *	0.79 (0.73–0.85) ***	0.7 (0.62–0.8) ***
Maternal age	1.1 (0.98–1.26)	1.17 (1.07–1.27) ***	1.02 (0.89–1.17)
BMI	1.65 (1.43–1.9) ***	1.44 (1.29–1.6) ***	0.72 (0.61–0.85) ***

ARTs: assisted reproductive techniques, BMI: body mass index, OR: odds ratio, CI: confidence interval. Current smoking: yes/no, smoking before pregnancy: yes/no, smoking cessation: yes/no. *: *p*-value < 0.05; ***: *p*-value < 0.001.

## Data Availability

Data are available upon request.
